# Dispersion Control over Molecule Cohesion: Exploiting and Dissecting the Tipping Power of Aromatic Rings

**DOI:** 10.1021/acs.accounts.3c00664

**Published:** 2024-03-27

**Authors:** Ricardo A. Mata, Tlektes Zhanabekova, Daniel A. Obenchain, Martin A. Suhm

**Affiliations:** Institute of Physical Chemistry, University of Göttingen, Tammannstrasse 6, 37077 Göttingen, Germany

## Abstract

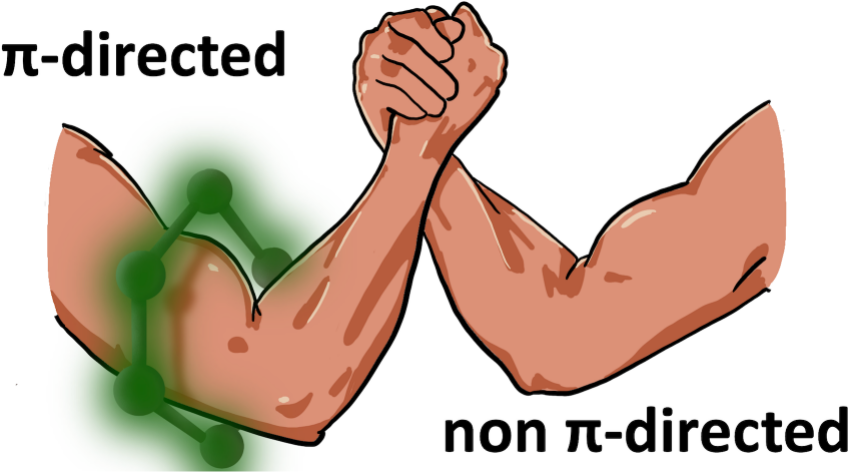

We have learned over the past years how London dispersion forces can be effectively used to influence or even qualitatively tip the structure of aggregates and the conformation of single molecules. This happens despite the fact that single dispersion contacts are much weaker than competing polar forces. It is a classical case of strength by numbers, with the importance of London dispersion forces scaling with the system size. Knowledge about the tipping points, however difficult to attain, is necessary for a rational design of intermolecular forces. One requires a careful assessment of the competing interactions, either by sensitive spectroscopic techniques for the study of the isolated molecules and aggregates or by theoretical approaches. Of particular interest are the systems close to the tipping point, when dispersion interactions barely outweigh or approach the strength of the other interactions. Such subtle cases are important milestones for a scale-up to realistic multi-interaction situations encountered in the fields of life and materials science. In searching for examples that provide ideal competing interactions in complexes and small clusters, aromatic systems can offer a diverse set of molecules with a variation of dispersion and electrostatic forces that control the dominant and peripheral interactions. Our combined spectroscopic and theoretical investigations provide valuable insights into the balance of intermolecular forces because they typically allow us to switch the aromatic substituent on and off. High-resolution rotational spectroscopy serves as a benchmark for molecular structures, as correct calculations should be based on correct geometries. When discussing the competition with other noncovalent interactions, obvious competitors are directional hydrogen bonds. As a second counterweight to aryl interactions, we will discuss aurophilic/metallophilic interactions, which also have a strong stabilization with a small number of atoms involved. Vibrational spectroscopy is most sensitive to interactions of light atoms, and the competition of OH hydrogen bonds with dispersion forces in a molecular aggregate can be judged well by the OH stretching frequency. Experiments in the gas phase are ideal for gauging the accuracy of quantum chemical predictions free of solvent forces. A tight collaboration utilizing these three methods allows experiment vs experiment vs theory benchmarking of the overall influence of dispersion in molecular structures and energetics.

## Key References

- WuttkeA.; FeldtM.; MataR. A.All that binds is not gold—The relative weight of aurophilic interactions in complex formation. J. Phys. Chem. A2018, 122, 6918–692510.1021/acs.jpca.8b0654630088931 .^1^*The balance between ligand and aurophilic/metallophilic interactions is discussed in detail for selected molecular crystals on the basis of calculations.*
- GottschalkH. C.; PoblotzkiA.; FatimaM.; ObenchainD. A.; PérezC.; AntonyJ.; AuerA. A.; BaptistaL.; BenoitD. M.; BistoniG.; BohleF.; DahmaniR.; FirahaD.; GrimmeS.; HansenA.; HardingM. E.; HochlafM.; HolzerC.; JansenG.; KlopperW.; KoppW. A.; KrasowskaM.; KrögerL. C.; LeonhardK.; Mogren Al-MogrenM.; MouhibH.; NeeseF.; PereiraM. N.; PrakashM.; UlusoyI. S.; MataR. A.; SuhmM. A.; SchnellM.The first microsolvation step for furans: New experiments and benchmarking strategies. J. Chem. Phys.2020, 152, 16430310.1063/5.000446532357787 .^2^*Meeting points between theory and experiment for the competition between hydrogen bonding and London dispersion are explored on the basis of a blind challenge.*
- ZimmermannC.; LangeM.; SuhmM. A.Halogens in acetophenones direct the hydrogen bond docking preference of phenol via stacking interactions. Molecules2021, 26, 488310.3390/molecules2616488334443471 PMC8400467.^3^*This work introduces aromatic solvents to the concept of intermolecular ketone balances, leaving them the choice to pick the aromatic side of the ketone or the nonaromatic side for their preferential docking.*
- Quesada-MorenoM. M.; SchnellM.; ObenchainD. A.Rotational analysis of naphthol-aromatic ring complexes stabilized by electrostatic and dispersion interactions. Phys. Chem. Chem. Phys.2022, 24, 1598–160910.1039/D1CP04337D34942639 .^4^*The structural balance of OH*···*π and OH*···*O binding motifs is examined by rotational spectroscopy of complexes with 1-naphthol, including furan.*

## 1 Introduction

The attraction between molecules is a sum of more or less directional competing forces, which can be physically dissected according to a number of different quantitative decomposition schemes.^5−9^ Chemists often like to identify primary interaction centers, such as hydrogen bond donors and acceptors or dispersion energy donors,^10^ and to construct qualitative networks of these local contacts. Quantum chemical concepts and visualizations which mediate between the global physical decomposition approaches and the local chemical functionalities meet with particular interest.^11−14^ In the end, hard experimental facts are needed to judge where the simplified models and concepts are productive and where they reach their limits.^15^ For noncovalent interactions, the spectroscopy of cold molecular complexes is a valuable source of experimental data,^16,17^ complemented by the structurally more accessible but computationally and conceptually more demanding solid-state limit. Here, we describe how rotational and vibrational spectroscopy in the gas phase can impose such experimental constraints on selected competing interactions. As our common motif, we choose the introduction of aromatic substituents on top of a classical hydrogen bond or aurophilic interaction. The typical goal is to trigger a switch in aggregation preference, induced by the London dispersion power^18^ of the aryl groups.

**Figure 1:**
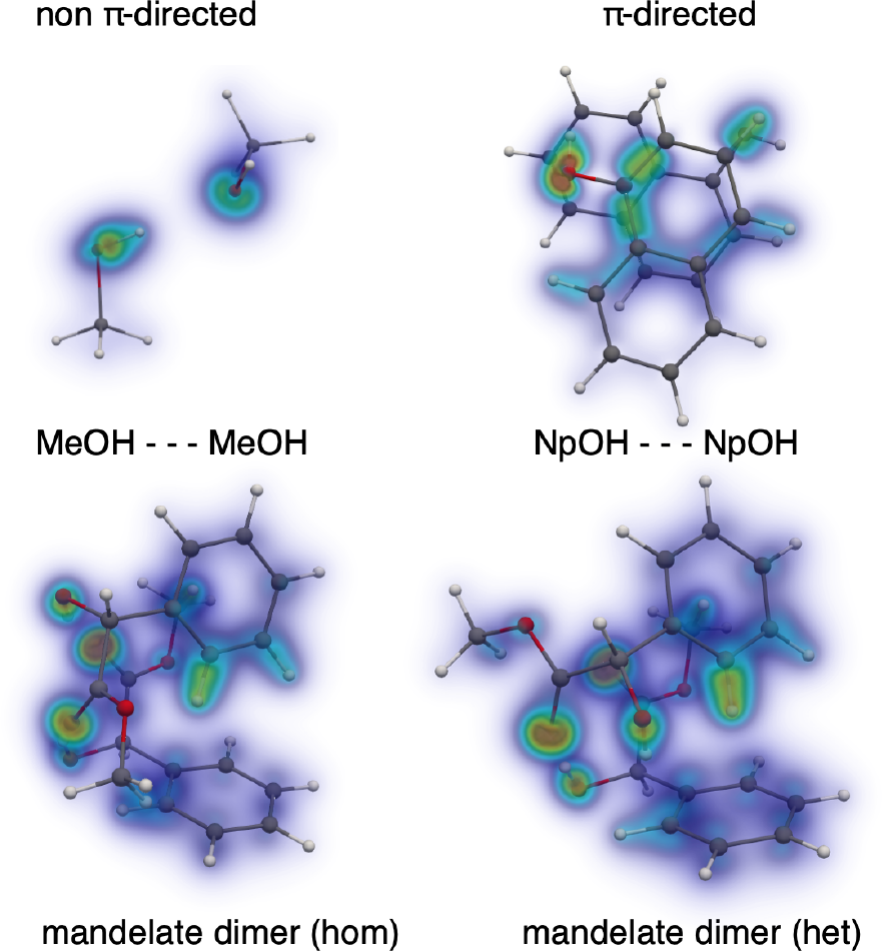
DID plots of selected molecular pairs. These highlight electronic densities in each monomer which interact significantly through dispersion with the other paired monomer. Weak dispersion interactions are represented in blue, up to green, and ultimately red for strong interactions. Dispersion switches from a secondary to a primary role in cohesion when moving from methanol (MeOH) to naphthol (NpOH). From homochiral to heterochiral methyl mandelate dimers, a classical hydrogen bond is sacrificed in favor of weaker OH···π and CH···π interactions, but the total cohesion still increases.

Sometimes, very subtle changes in a molecule can drastically modify its aggregation behavior. A particularly pronounced case is the tetramerization of lactates, where the switch from homochiral to racemic composition triggers a drastic reorganization of the hydrogen bond topology.^19^ The four lactate units can move closer together when left- and right-handed copies alternate and at the same time the four OH groups give up their disk-like, cooperative hydrogen bonding in favor of densely packed but isolated hydrogen bridges to C=O. The number of classical hydrogen bonds is thus preserved, but cooperativity loses against London dispersion and the effect vanishes when long-range dispersion is left out of the modeling. It is fairly unusual that the mere swapping of H and CH_3_ at a stereogenic center of a highly flexible molecule such as methyl lactate has such a large effect on the hydrogen bond pattern in its tetramer. For systematic studies, it is advisable to explore aromatic substituents as stronger dispersion energy donors. Substitution of the methyl groups^19^ by phenyl units at the chiral center of lactates leads to mandelates^20^ and introduces new enantioselective aggregation patterns already at the level of dimers. Even the number of classical OH···O hydrogen bonds is modulated by the relative stereochemistry of the aromatic substituents (Figure 1).^20^ When the two mandelate units have the same chirality (hom), they realize two classical OH···O=C hydrogen bonds and at the same time manage to bring their phenyl groups together via dispersion interactions. For opposite chirality (het), this does not work anymore, and instead of giving up the aromatic dispersion interactions, one of the classical hydrogen bonds is sacrificed and replaced by additional or stronger dispersion-dominated OH···π and CH···π contacts (Figure 1). The net result is a gain in cohesion by about 10% despite the loss of a strong hydrogen bond. In the limit of the solid state, the hydrogen bonds generate infinite chains and the aromatic groups no longer compete with this hydrogen bonding but rather cooperate, at the same time controlling their interlock in quite unusual stereochemical sequences.^20^ Other cases of enantioselectivity mediated by aromatic London dispersion have been studied^21,22^ and illuminate the diversity of chirality control in supramolecular chemistry.

The influence of aromatic rings on the docking preferences of achiral molecules is equally diverse.^23,24^ All of these experimental studies contribute to the benchmarking of computational methods^15^ because they involve subtle energy differences which can be experimentally decided in favorable cases, at least in the sense of an energy order between competing conformations.^25^ Note that the introduction of an aromatic ring has several consequences beyond adding more dispersion interaction possibilities, such as inductive through-bond effects or steric hindrance. There are several theoretical techniques available to visualize and help interpret such competing factors. For this publication, we resort to plots of dispersion interaction densities (DIDs), Figure 1),^13^ which are wave function-based and prove useful for observing how different moieties contribute through London forces. The DIDs are a visualization of weighted local orbital densities. The dispersion energy is a sum of pair correlation energies for two occupied orbitals localized in different monomers. For the sake of simplicity, we are considering the case of a dimer (A–B), with occupied orbital *i* in monomer A and orbital *j* in B, and restricting the discussion to the closed-shell MP2 level of theory
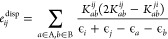
1

The respective orbital densities are then weighted as
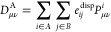
2with *P^i^* being the local orbital *i* density and resulting in the DID matrix for monomer A. This can then be represented in real space with the use of voxels. Large values correspond to electronic densities that strongly interact through dispersion with another molecule.

The use of aromatic rings to control the way in which molecules aggregate has additional benefits on the technological side. The relatively rigid, planar scaffolds do not introduce unnecessary dynamical complexity into the microwave spectra, from which the structure of the aggregates can be obtained. The availability of aromatic UV chromophores invites size- and conformationally selective double resonance vibrational spectroscopy techniques.^26−31^ Nevertheless, we will make heavy use of linear vibrational spectroscopy^32,33^ because, like microwave spectroscopy, it allows us to study the nonaromatic counterparts on an equal footing for the best comparison. Aromatic chromophores are also interesting because they enable the experimental determination of dissociation energies in favorable cases. Theoretical predictions can then be judged based on absolute values.^34,35^ In the absence of such absolute values, the interconversion between energetically similar conformations in a supersonic jet at least provides relative energy sequences if the barriers between them are efficiently overcome in supersonic expansions.^36−38^

## 2 Representative System Classes

In the following, aromatic substituents will be abbreviated as Ar and the chemically attached functional groups (largely OH in this work) will be highlighted by connecting them with a dash. We discuss directly attached OH groups, more flexibly attached OH groups, and as a final case the nondirected metallophilic interactions.

### 2.1 Aromatic Hydroxy Compounds (Ar–OH)

When switching from the simplest pair of alcohols, a methanol dimer (Figure 1),^33,39^ to the simplest pair of aromatic hydroxy compounds, a phenol dimer,^27,40^ nothing changes in the connectivity. The hydrogen bond donor ability is greatly enhanced by aromatic conjugation, but the acceptor ability is simultaneously attenuated in phenol. Therefore, the hydrogen bond-induced vibrational downshift of the OH group increases by less than 15%^27,39^ from methanol to the phenol dimer. Furthermore, the rigid attachment of the aryl group to the OH docking unit and the directionality of the hydrogen bond both disable the aryl groups from optimizing their secondary dispersion interactions.^40^ When moving on to the cyclic homotrimers, the first qualitative change occurs. In contrast to methanol, where only two methyl groups point on one side of the ring,^33,41^ the dispersion interaction among the three phenyl groups moves all of them on one side and enforces *C*_*3*_ symmetry.^27,40^ The hydrogen-bonded downshifts are now even within 5% of the methanol homotrimer,^27,33^ underscoring the compensation of the modified OH donor and acceptor strengths and the fact that OH hydrogen bonding is not compromised very much by dispersion interactions between the different hydrocarbon residues attached. Vibrational spectroscopy is clearly not the most sensitive marker for aromatic substitution effects in such a case. Things become more interesting when moving to 1-naphthol, which comes in two conformations. Based on IR spectroscopy,^31^ the most stable homodimer was found to be dominated by dispersion forces, leading to π···π stacking (Figure 1) with a little bit of secondary O–H···O hydrogen bonding. A microwave investigation^42^ later even disproved this small residue of classical hydrogen bonding, so the switch to dispersion control is actually rather complete.

**Figure 2:**
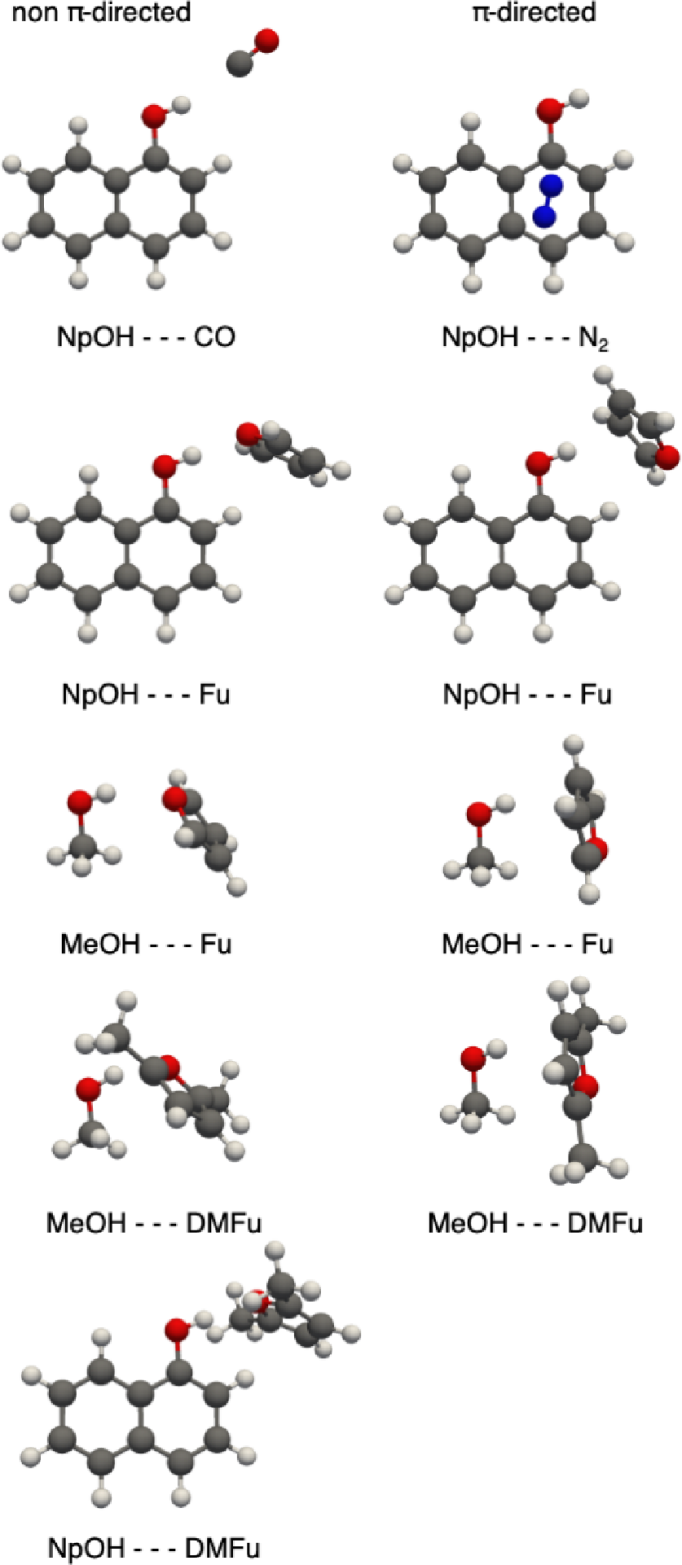
Competition between structures with classical hydrogen bonds (left) and π-directed dimer structures (right) for the different combinations of 1-naphthol and methanol with furan and dimethylfuran as well as contrasting docking preferences of CO and N_2_ on 1-naphthol.

If one wants to explore tipping points more closely, it is advisible to move away from homoaggregates toward heteroaggregates (Figure 2). After a major effort in characterizing the docking of methanol to furan and its derivatives in the form of a blind challenge for theory groups,^2,43^ it made sense to see what happens when methanol is replaced by 1-naphthol.^4^ This choice was further supported by the availability of absolute binding energies for these complexes^44^ as part of a larger series of 1-naphthol binding affinities by the Leutwyler group.^34,45^ As a binding partner, the furan motif was again utilized, having shown the potential to act as a classical hydrogen bond acceptor, as in the complexes with HF^46^ and H_2_O,^47,48^ but alternatively offering its π system, as was observed in the rotational studies of HBr,^49^ ethene,^50^ and acetylene.^51^ Coexisting OH···O and OH···π motifs have been explored extensively for methanol, and the OH···O motif wins by a tiny energy margin.^2,43^ Would that coexistence survive if methanol is replaced by 1-naphthol? In the determination of the dissociation energy of the 1-naphthol···furan complex by the Leutwyler group, two possible structures were proposed based on DFT predictions but could not conclusively be identified in the SEP-R2PI laser method. The microwave spectroscopy study confirmed two distinct structures quite analogous to those of methanol (Figure 2), one with an OH···O bridge and a weaker CH···π interaction and the second structure with a primary OH···π interaction together with a stabilizing CH···O contact. Experimental structures^4^ support an overall *C*_*s*_ symmetry in both observed complexes, while the DFT results of the Leutwyler group showed a mix of *C*_1_ and *C*_*s*_. This demonstrates the need for accurate structures when benchmarking gas-phase complexes. It also shows that the effect of aromatic rings can be minor when the modulation of dispersion interactions is counteracted by a change in OH donor ability due to aromatic conjugation. Changing the acceptor ability of furan by adding two methyl groups (DMFu, Figure 2) has little consequence for methanol as a donor, but for naphthol, the secondary conformation with the OH···π bond remains elusive.

Anisole is another molecule which offers two closely competing binding sites to an OH group, namely, the exocyclic oxygen atom or the aromatic ring itself. Other than for furan, the interconversion barrier is somewhat broader because it requires donor motion across the ring boundary. For both methanol^52^ and 2-naphthol^53^ as binding partners, theory is quite undecided about the docking preference, but in experiments, only the oxygen docking isomer is observed.^52,53^ Considering the large change in donor character from methanol to 2-naphthol, this experimental uniformity, despite close competition, is again quite surprising. It is expected that relatively minor further modifications of the anisole can tip the balance in favor of π bonding.

The long series of binding energies^45,54^ for the more extensively studied 1-naphthol donor contains further interesting ambiguities concerning the docking preference of acceptor molecules. Compared to the subtle geometry issues in the complex with furan,^4^ a much larger difference was observed for carbon monoxide. In the original work of the Leutwyler group,^55^ experimental dissociation energies agreed well with the DFT predictions such that the carbon monoxide complex was assumed to be interacting with the aromatic rings of 1-naphthol, by analogy to the N_2_ case (Figure 2).^44,56^ This was done despite a substantial increase in binding in the S_1_ electronically excited state, which would be more typical for hydrogen bond coordination to the OH group. In a more recent work,^57^ we used rotational spectroscopy to show that the complex is in fact hydrogen bonded and has no mass out of the plane of the 1-naphthol, making it unambiguously *C*_*s*_-symmetric (a so-called “edge” structure, Figure 2). This was the only complex with carbon monoxide observed. Within the experimental error bar, the experimental dissociation energy (*D*_0_ = 7.68 ± 0.84 kJ mol^–1^) still agrees with the predicted value at the time of 8.07 kJ mol^–1^ (B97-D3/QZVPP).^55^ To resolve this structural indeterminacy also on the theory side, we recomputed the binding energies for the conflicting structures of 1-naphthol and carbon monoxide using the PNO-LCCSD(T)-F12 approach with extrapolation to the CBS limit. (Further details are provided in the SI.) The results favor the edge structure in agreement with the microwave results, predicting a dissociation energy of 8.13 kJ mol^–1^ (compared to 7.24 kJ mol^–1^ for the face structure). Thus, the 1-naphthol structurally matches smaller hydrogen bond donors such as water^58^ or phenol^59^ when binding to CO, whereas the extra aromatic ring is able to swap N_2_ from hydrogen-bonded in the phenol complex to aromatic ring- or dispersion-bound in the 1-naphthol complex.^56^

Moving from diatomics to more extended π acceptors, ethene offers a particularly simple scaffold. The measured 1-naphthol···ethene complex still appears to prefer an OH···π geometry,^54^ while three chlorinated derivatives of ethene (*trans*-1,2-dichloroethene, trichlorethene, and tetrachloroethene) favor π···π interactions on the face of 1-naphthol. Rotational spectroscopy studies confirm that ethene coordinates perpendicular to the planar 1-naphthol,^57^ which allows for torsional freedom. The high-resolution rotational spectrum still suggests that the ethene tunneling motions are quenched. When replacing 1-naphthol by methanol, this may no longer be the case for several reasons. The binding energy will be further reduced from the 13.37 ± 0.29 kJ mol^–1^ observed for 1-naphthol,^54^ ethene rotation around the OH···π bond is nearly barrierless,^60^ and the methyl group introduces another soft rotor. Despite this floppiness, the vibrational signature of the methanol–ethene complex is deceptively simple^60^ and matches theoretical predictions for the hydrogen bond-induced shift, indirectly validating these predictions in terms of the dissociation energy and low barrier. Still, it will be rewarding (but perhaps nontrivial) to check the good match between vibrational spectroscopy and theory by using rotational spectroscopy to directly probe the large-amplitude motions in methanol–ethene.

If the binding partner of 1-naphthol can also act as a hydrogen bond donor, then a new kind of large-amplitude motion emerges. The two OH groups may then interchange their donor–acceptor roles. For 1-naphthol in combination with water, there is a clear preference for water acting as the acceptor.^61^ Moving to the nonaromatic methanol–water complex, water instead preferentially acts as a donor.^62^ However, this may be attributed predominantly to the higher acidity of the aromatic hydroxyl group rather than to dispersion interactions.

**Figure 3:**
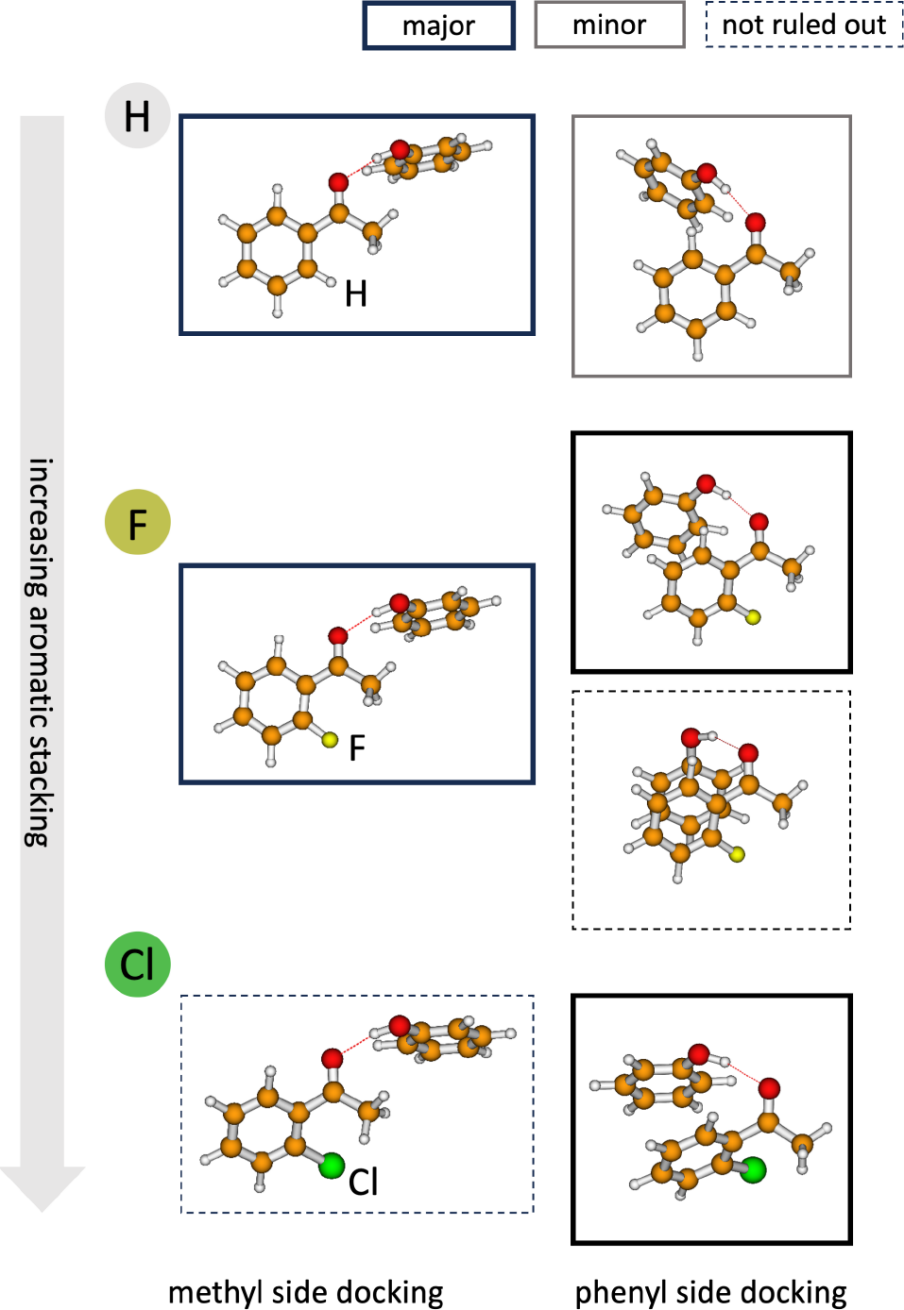
Phenol can add to the carbonyl group of acetophenone either on the methyl side (left) or on the phenyl side (right), and the experimental preference (frames) is switched from methyl to phenyl docking by replacing the aromatic C–H in the 2-position by C–F or C–Cl. The driving force for this intermolecular balance is stacking of the aromatic rings, which is facilitated by the nonplanarity of 2-substituted acetophenones. The structures are classified according to their estimated population in the jet experiment, as major, minor, or as not ruled out.

A tipping experiment which avoids any switch in the donor–acceptor roles has been designed for acetophenone (Figure 3). Acetophenone offers two nearly equivalent attractive hydrogen bond acceptor sites: the two lone electron pairs of the C=O group.^3^ One points in the direction of the phenyl ring and thus has a higher potential for London dispersion interactions. To compensate for this intrinsic advantage, the phenyl group includes a CH bond in the ortho position which points in the direction of that lone electron pair and thus enforces a suboptimal hydrogen bond angle for a docking donor. The other lone electron pair points in the direction of the almost freely rotating methyl group, which can avoid the CH geometrical restriction by facile torsion. To compensate for the steric advantage of the methyl docking side, methyl groups undergo fewer dispersion interactions with donors than phenyl groups. Besides these fine details, the local environments are rather similar for the two C=O lone electron pairs, and thus one can hope that the zero-point vibrational energy, which frequently prevents a direct comparison of electronic structure predictions with experimental docking preferences, largely cancels between the two sites. Therefore, so-called ketone intermolecular balances^25^ are more favorable for a straightforward theory–experiment comparison than the previously discussed furan docking balances^2^ as long as the interconversion barrier is not much higher than the one between carbon and oxygen docking in a furan ring.

It was therefore of interest to compare methanol^3,25^ and phenol^3^ docking to the ketone group of acetophenone. Methanol unambiguously prefers the sterically less demanding methyl docking site, and the same is still true for phenol because the phenyl ring of the donor is able to position itself halfway in the direction of the phenyl group of acetophenone for some supporting π–π stacking and further London dispersion interaction with the ketone.^3^ The less stable conformation based on the phenyl-side docking of phenol, which is observed as a trace for regular acetophenone, is clearly preferred when the C–H group in the other ortho position of acetophenone is replaced by C–Cl or C–Br. C–F in the ortho position brings the system close to the structural tipping point, and actually there is now some vibrational spectroscopy evidence for three different species, methyl-side docking, phenyl-side hydrogen bonding, and phenyl-side stacking at the expense of the hydrogen bond strength (Figure 3).^3^ This and the problems in reproducing the close balance by standard quantum chemical methods clearly call for a microwave study to unambiguously resolve the competing phenol–acetophenone complex structures, but London dispersion is already seen to tune the preference at the keto group from one side to the other when assisted by halogenation.

When moving from ketones to α-hydroxyesters, competition with their intramolecular hydrogen bond comes into play. Replacing methanol^63^ by phenol^64^ as a binding partner increases the number of experimentally observed hydrogen bond patterns, but this effect will be discussed in more detail for benzyl alcohol.^65^

In summary, the rigidity of the phenol scaffold limits the amount of London dispersion leverage in its complexes so that additional measures such as halogenation and annulation (to naphthol) are frequently needed for major changes in the docking preference relative to methanol.

### 2.2 Aromatically Substituted Methanols (Ar–CH_2_–OH)

Inserting a CH_2_ group between the aromatic ring and the OH group of phenol has two major effects. It interrupts the π conjugation, turning the OH group into a typical alcohol group, and it gives more flexibility to London dispersion forces from the aromatic ring which compete with the primary hydrogen bond option. Therefore, it is particularly instructive to compare benzyl alcohol self-aggregation with that of methanol or ethanol. Although benzyl alcohol has several conformational options, only one pair of enantiomers is relevant at low temperatures. They synchronize torsion around the CO bond and around the CC bond to gauche or synclinal angles of the same handedness, so in a sense they represent enantiomeric helices which can easily interconvert by tunneling or thermal excitation.^66^ Therefore, the homodimer comes in a homochiral and a heterochiral variant. The homochiral pairing wins by a small (<2 kJ/mol) margin. Both structures have the same primary hydrogen bond topology as the methanol dimer, but the free OH group undergoes weak, dispersion-driven, secondary hydrogen bonding to the phenyl ring of the other unit. Only slightly higher in energy, a new binding motif is enabled by the aromatic ring, where the classical OH···O hydrogen bond is completely sacrificed in favor of two dispersion-driven OH···π bonds. Again, there is a homochiral and a heterochiral variant, but only the heterochiral variant is partially protected from relaxation into the more stable classical arrangement. For these conclusions, a combination of IR and Raman spectroscopy was essential due to the complementary transition moments.^66^ Comparison to popular quantum chemical calculation shows that none of the employed density functionals are able to reproduce the energetics and the spectra in a fully satisfactory manner. There is thus room for improvement in the balanced description of electrostatics and London dispersion.

For the homotrimer and homotetramer of benzyl alcohol, a rare multimessenger analysis of microwave, linear infrared, spontaneous Raman, and IR/UV double resonance spectroscopy^67^ has revealed a much more complex conformational landscape than for the methanol trimer and tetramer.^33,41^ The switch from open to cyclic patterns of classical hydrogen bonds is delayed compared to methanol because hydrogen-bonded ring strain can be released through OH···π contacts. The homochiral preference found in the dimer is probably recovered for the tetramer and continues into the solid state.^67^

As in the case of phenols, tuning possibilities increase by moving to heterodimers. Replacing one aromatic ring of the benzyl alcohol dimer with the hydrogenated cyclohexyl variant^68^ strongly favors the remaining benzyl alcohol as the hydrogen bond donor, partially because otherwise there would be no dispersion-driven OH···π back-donation option for the acceptor. However, it leaves an interesting dilemma between a structure which realizes this back-donation and a structure which resembles a methanol dimer but exploits an almost perfect parallelism of the phenyl and cyclohexyl groups, resulting in a dispersion interaction between the aliphatic and aromatic rings. The spectra indicate that the latter ring–ring dispersion actually wins, and this is marginally confirmed at a high electronic structure level including zero-point energy corrections.^68^ It would be valuable to check this vibrational spectroscopy finding by rotational spectroscopy. The complex might be considered to be a spectroscopically viable gas-phase model system for graphene–graphane interactions.^69^

Smaller binding partners of benzyl alcohol show interesting differences from the case of methanol binding. Toward water, benzyl alcohol acts as a hydrogen bond donor.^28,70^ This is again due to the possibility of the water acceptor interacting with the aromatic ring. Such a secondary interaction is not possible for methanol, which therefore acts as a hydrogen bond acceptor toward water^62^ following its intrinsic preference. The same situation is found for 1-phenylethanol^29,30^ in comparison to ethanol.^62^

**Figure 4:**
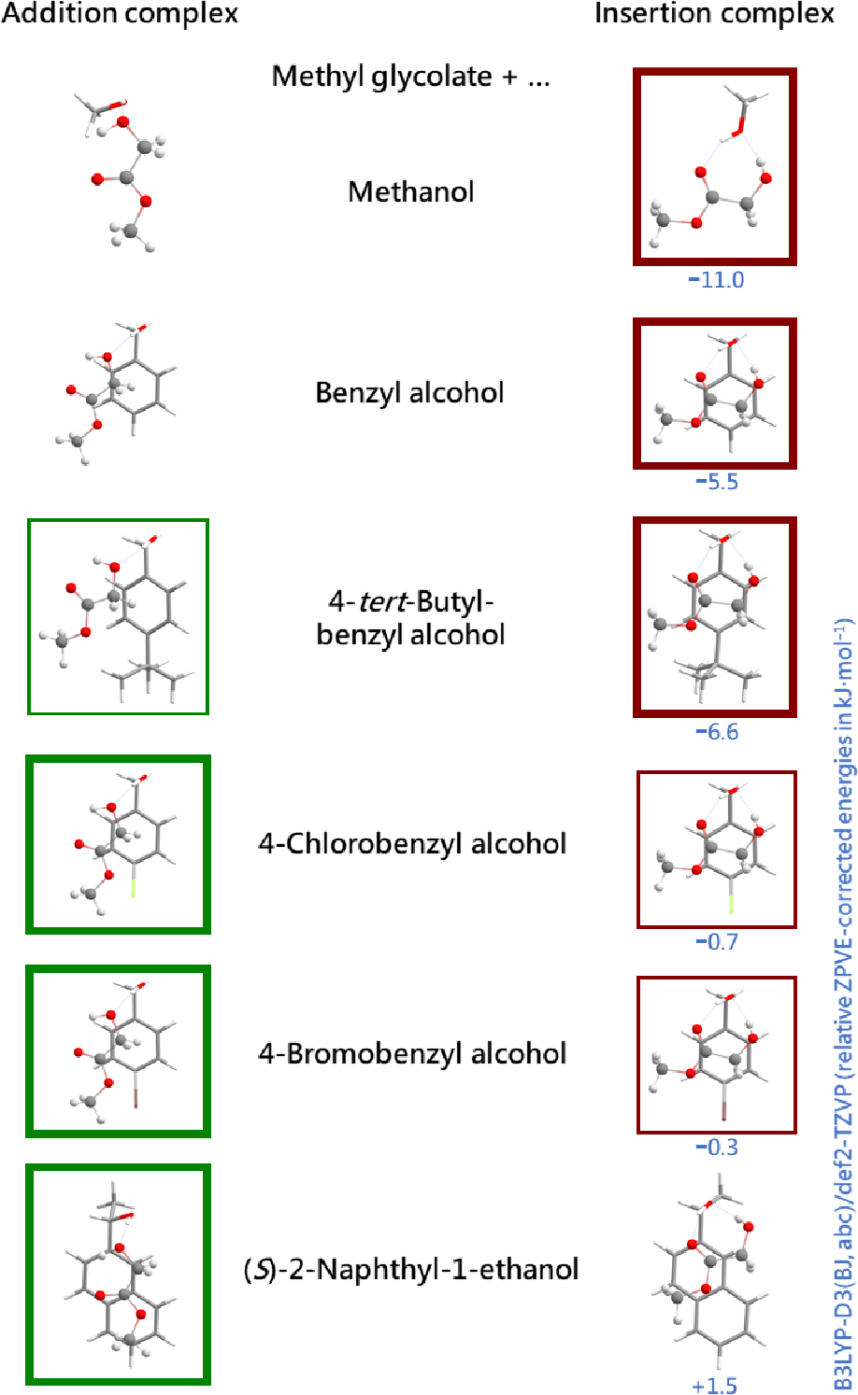
Alcohols can either add to the internal hydrogen bond of methyl glycolate (left) or insert into it (right). With increasing residue size, the addition complex (green frames) gains in the competition with the insertion complex (red frames). The switch happens somewhat earlier in the experiment than predicted at the harmonic DFT level (blue). See the text for further explanations.

A more complex situation arises when alcohols interact with α-hydroxyesters such as methyl glycolate (Figure 4) and methyl lactate because the experimental preparation of the binary complexes relies on sufficiently low barriers to insertion of the alcohol into the intramolecular hydrogen bond of the ester if this corresponds to the most stable arrangement. Methanol clearly succeeds in inserting and thus in reaching the most stable conformation.^63^ Benzyl alcohol does the same for methyl glycolate, but for methyl lactate, most of the observed complexes avoid insertion, although the insertion process should be slightly downhill.^65^ This is also due to the dispersion interaction of the phenyl ring with the hydroxyester, which raises the barrier for interconversion. By adding a *tert*-butyl group in the para position, the barrier is further increased and now the insertion is already incomplete for methyl glycolate.^71^ Replacement of the *tert*-butyl group by a halogen (Cl, Br) also blocks insertion more or less completely but now insertion is thermoneutral or uphill (Figure 4), so this is more expected. A comparison of DFT predictions to CCSD(T) energies indicates some small but systematic errors in the former, also based on experimental findings.^71^ As Figure 4 suggests for the glycolate case, a shift in computed relative energies on the order of a few kJ mol^–1^ may already explain the experimental preferences without having to invoke blocking barriers. This ambiguity needs further investigation. Extension of the aromatic substituent to naphthyl also suppresses insertion (Figure 4), and together with an extra methyl group it generates chirality discrimination.^72^ Halogenation of the phenyl group in the ortho position is expected to introduce further interesting effects.^73^ Control of the reaction barrier heights in low-temperature supramolecular reactions by London dispersion interaction is a design strategy which may be transferable to room-temperature reactions if the barriers are made higher.^71^

In summary, the higher flexibility of benzyl alcohol enables an earlier manifestation of competing London dispersion interactions. The conformational landscape becomes more corrugated, which poses challenges for the theoretical structure and conversion path search as well as for the experiment to reach the lowest-energy minimum.

### 2.3 Aromatically Substituted Gold Compounds (Ar–Au–X)

Understanding the competition between intermolecular forces can also prove crucial in rationalizing the function and structure of coordination complexes. A particular example of this discussion is centered on aurophilic interactions. Gold is often used in catalysts and molecular interactions may influence activity/selectivity, and there are many questions revolving around the structure of molecular crystals. It has been known for quite some time that cationic closed-shell d^8^ or d^10^ metal complexes can build stable dimers, despite the Coulomb repulsion between the metal centers. The most notable example is Au^I^, albeit other metals can also participate such as Ag^I^, Hg^II^ (d^10^), and Rh^I^ (d^8^). This has led to the term metallophilic interaction, in general. The theoretical investigations that first helped to characterize these interactions were based on relatively small molecular complexes,^74−77^ wherein the dominant stabilizing forces involved the Au^I^ centers. Over the years, this has led many authors to the conclusion that close Au^I^–Au^I^ contacts (around or even below 4 Å) would be foremost caused by the aurophilic effect. Similar assumptions were made for other close metal contacts. What was oftentimes left out of the picture was the impact of ligand–ligand interactions. This would change over the years as the impact of dispersion interactions was studied in further detail.^18^

Gas-phase data on aurophilic/metallophilic complexes is scarce.^79^ Most of the studies are of a computational nature or involve experimental investigations of molecular crystals. On the theory side, several studies decomposed the interactions of selected complexes, but these would be generally small.^80,81^ This was linked with the computational cost, as both electronic correlation and relativistic effects have to be included to quantitatively assess aurophilicity. As such, the modeled ligands were not large enough or functionalized, and the Au–Au (or more generally metal–metal) interactions would outweigh other contacts. Only in 2013 did we see the first analyses of competing effects.^78^ The conclusions were that the dispersion forces linked to the gold centers would be sizable only below a Au–Au distance of 3.7 Å and that aryl interactions could outweigh the aurophilic interaction. Similar conclusions had already been drawn in a work by Grimme and Djukic,^82^ where the authors observed that in a Rh^I^–Rh^I^ complex dimer association was mostly driven by aryl–aryl interactions, not by the interaction of the two close-lying metal centers.

**Figure 5:**
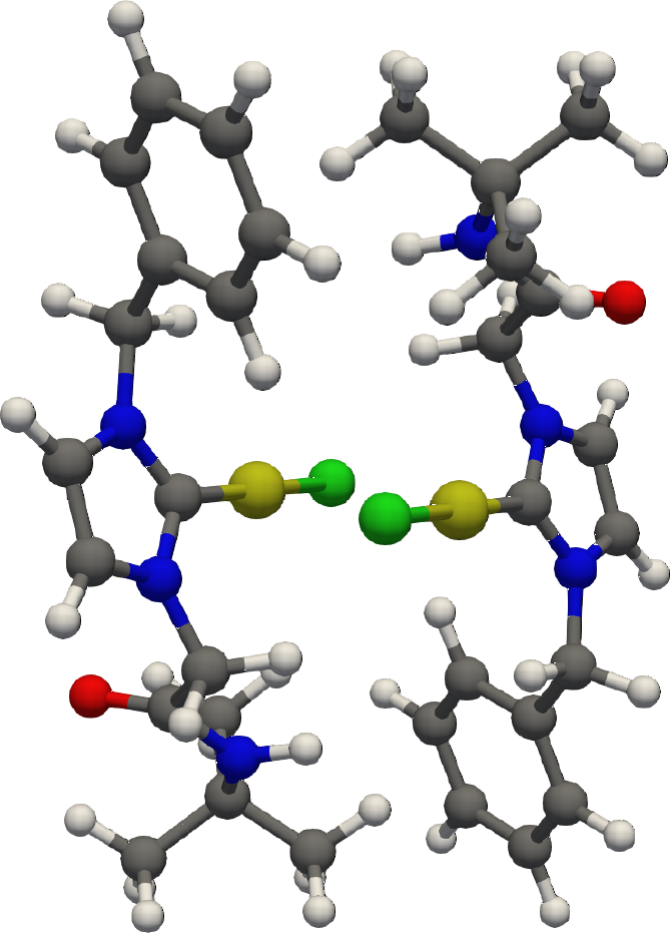
Representation of the [1-(benzyl)-3-(*N*-*tert*-butylacetamido)imidazol-2-ylidene]AuCl_2_ dimer complexes observed in the solid state by Ghosh and co-workers, as discussed in the text and computed in ref (78).

A similar example can be taken from the Au^I^ and Ag^I^ dimer complexes synthesized and characterized by Ray et al. (Figure 5).^83^ The authors made the somewhat counterintuitive observation that the Ag^I^–Ag^I^ interaction would be stronger than the Au^I^–Au^I^ interaction, given that the distance in the dimer increased when swapping the metal from Ag to Au (3.197 vs 3.204 Å). This was a counterintuitive finding but was in line with an earlier theoretical study which argued that the metallophilicity would decrease from Ag to Au.^84^ Both studies failed to recognize that due to the smaller vdW radius of Ag^I^ compared to Au^I^, the ligands could come into closer contact for the Ag dimers. This resulted in an overall stronger interaction. In a more recent publication,^85^ the evolving views on the competition between metallophilicity and ligand interactions have been compiled and discussed. When addressing the structures of these complexes, particular care should be taken, considering the metal–metal distances but also other contacts present. A rather straightforward case is provided by the molecular crystals of [Au(Me_2_bimy)Cl] and [Au(Et_2_bimy)Cl]. Albeit one observes close Au^I^–Au^I^ contacts, the stabilization provided by the aryl interactions outweighs the metal–metal contributions.^1^ Just like in the case of hydrogen-bonded dimers, the competitive effect of aryl moieties can overcome other interactions given their large surface and number of contact points. Dispersion adds up just as highlighted in Figure 1. Authors have generally become more aware of these issues as of late.^86^ The reason that aryl interactions are sometimes neglected becomes even clearer with these examples. A hydrogen bond or two metal centers coming close together draws more attention or is easier to rationalize than the multitude of contacts that needs to be tallied in the case of an aryl moiety. Visualization techniques such as DIDs have helped with the task, but our chemical intuition also requires further work.

## 3 Conclusions

In this Account, we discuss how the interactions of aromatic rings compete with strong noncovalent directed (OH) and nondirected (metallophilic) interactions. Direct, rigid aromatization of alcoholic OH groups leads to a number of effects on molecular aggregation preferences. Improved through-bond hydrogen bond donor and decreased hydrogen bond acceptor abilities tend to compensate for each other. The added through-space London dispersion modulates the hydrogen-bonded network and can dominate the interaction for bicyclic aromats.

A methylene linker between the aromatic substituent and the OH group is less disruptive to the hydrogen bond donor and acceptor properties, and the increased flexibility allows for the release of ring strain in cyclic aggregates. Benzylic substitution can increase barriers for isomerization due to its secondary interactions and thus may prevent annealing to the lowest minimum structures of molecular complexes in their supersonic jet synthesis, but an extensive characterization of the potential energy landscape is required for firm conclusions.

In the solid state, some of the dispersion effects observed in finite aggregates are attenuated because ring topologies with their implicit strain often relax to infinite chains and the competition between London dispersion and hydrogen bonding eases. Nevertheless, some of the dispersion-driven homochirality propensities in finite clusters anticipate the preference in the solid state. In any case, a theoretical method which is unable to predict structural preferences for small clusters is unlikely to perform well for the infinite solid, at least for the right reason. Therefore, it would be best to routinely complement solid-state calculations by those of small fragments, where comparison either to gold standard calculations^87,88^ or even better to a gas-phase experiment^15^ is then made to provide a measure for the expected error. One clearly observes the importance of converging the level of theory in so-called metallophilic complexes. Conclusions based on the geometry alone might be erroneous, as aryl–aryl or aryl-metal interactions easily add up. Albeit the electronic cloud of an aryl carbon is much smaller than the individual metal centers, aryl contacts can easily dominate due to the sheer number of contacts.

## Supporting Information Available

Further computational details, calculated dissociation energies for the 1-naphthol···CO system, and molecular structures (in *xyz* format) calculated for this work (PDF)
