# Supplementary material for: Dispersion Control over Molecule Cohesion: Exploiting and Dissecting the Tipping Power of Aromatic Rings
Source: Acc Chem Res. 2024 Mar 27;57(8):1077–86. doi: 10.1021/acs.accounts.3c00664 (PMC11025128; doi:10.1021/acs.accounts.3c00664)
Supplement: Supplementary file 1 — ar3c00664_si_001.pdf [file ar3c00664_si_001.pdf]

Supplementary information: Dispersion control over molecule cohesion: Exploiting and dissecting the tipping power of aromatic rings

Ricardo A. Mata,* Tlektes Zhanabekova, Daniel A. Obenchain,* and Martin A. Suhm*

Institute of Physical Chemistry, University of Göttingen, Tammannstrasse 6, 37077, Göttingen, Germany

E-mail: rmata@gwdg.de; daniel.obenchain@uni-goettingen.de; msuhm@gwdg.de

1 Computational details

The minimal energy structures and the harmonic vibrational frequencies of the 1-naphthol complexes were computed at the B2PLYP-D3(BJ) level of theory,[1] using the D3 Grimme dispersion correction,[2] Becke–Johnson damping[3] and the def2-TZVPP basis set.[4] The Gaussian 16A.03 program package was used for the latter calculations. For the 1-naphthol-CO complex we also reference the binding energy D_e (Table S1). The latter was computed by recalculating the electronic energy at the PNO-LCCSD(T)-F12[5–9] level of theory using cc-pVDZ-F12 and cc-pVTZ-F12 basis sets[10] with the Molpro2022.2 program package.[11–13] The extrapolation to the CBS limit was calculated using the explicitly correlated CCSD-F12b correlation energies using the formula of Hill *et al.*[14]

Table S1: Calculated binding D_e and dissociation D_0 energies in kJ mol^{-1} for 1-naphthol-CO edge and face structures.

complex	VDZ-F12		VTZ-F12		CBS	
	D_e	D_0	D_e	D_0	D_e	D_0
edge	10.67	7.89	10.80	8.02	10.91	8.13
face	7.98	6.46	8.49	6.97	8.76	7.24

The remaining structures mentioned in the manuscript have been taken from the respective cited publications. The DID plots[15] were computed at the PNO-MP2/cc-pVTZ[16] level of theory.

2 Molecular structures

The structures below are taken from a more extended study of 1-naphthol dimer structures, which is due to be published. Here we provide already the B2PLYP-D3(BJ)/def2-TZVPP structures of the structures mentioned in the text. All other structures can be taken from the respective publications.

```
19
1-naphthol monomer
O       1.6610023901    -2.0979078543   -4.6179e-6
H       2.6165612715    -2.1985981594   -6.218e-6
C       1.3550110164    -0.7667399266   -3.1553e-6
C       -0.0273331325   -0.4370297157   -2.191e-7
C       2.3140593747    0.2176173669    -4.4798e-6
C       -1.0348355619   -1.4292520783   1.2225e-6
C       -0.3947843037   0.9405859531    1.2874e-6
C       1.9358227301    1.5761113271    -2.9331e-6
H       3.3627913497    -0.0515651482   -6.793e-6
```

C	-2.3606904038	-1.0717811559	4.0361e-6
H	-0.7434319188	-2.4682703048	8.04e-8
C	-1.7716821243	1.269185859	4.1754e-6
C	0.6132858745	1.9357578677	-1.054e-7
H	2.7057555029	2.3343971441	-3.9922e-6
C	-2.7322556465	0.2888263014	5.5014e-6
H	-3.1251678198	-1.8355667082	5.1299e-6
H	-2.0547646471	2.3132368896	5.3143e-6
H	0.3240221347	2.9772939772	1.0518e-6
H	-3.7790380864	0.5579663656	7.7146e-6

21

1-naphthol-CO complex edge

C	2.9051875647	1.5263512562	-0.0008119393
C	3.6533322318	0.3306739209	0.0061498542
C	3.0159698265	-0.8849146874	0.009204606
C	1.6026965525	-0.9673623046	0.0054691796
C	0.8533391588	0.2455476814	-0.0015654934
C	1.5325150946	1.4858087996	-0.0045990309
H	4.7333213382	0.3746220174	0.0090809828
H	3.5880545548	-1.8030722896	0.0145435193
H	0.9529561673	2.3960605742	-0.0099277995
H	3.4169190568	2.4782131179	-0.0031675285
C	-0.5666083111	0.1665807823	-0.0053340643
C	-1.2001666748	-1.0546720615	-0.0022464213
C	-0.4459266715	-2.2458823987	0.0047155176
C	0.9243758423	-2.2108075948	0.0085182271

H	-0.9647651635	-3.1940395858	0.0070349368
H	1.5010966363	-3.1250966913	0.0138548899
H	-2.2810724296	-1.0996954666	-0.0051784799
O	-1.2403156898	1.3501161225	-0.0120333131
H	-2.1894703882	1.183074202	-0.014079844
C	-4.4532319541	1.30330813	-0.0209283845
O	-5.5709731917	1.4539776585	-0.0246389438

21

1-naphthol-CO complex face

O	-1.3009483892	2.371183408	0.1615224557
H	-2.2368658031	2.5724747026	0.0788122235
C	-1.0683668351	1.1225293413	-0.3360581598
C	0.2649495569	0.6417496796	-0.2350606797
C	-2.0543088034	0.3494502791	-0.9022787171
C	1.293981292	1.4082525215	0.3591673729
C	0.5558306462	-0.6602545417	-0.737514574
C	-1.7518952504	-0.9360723761	-1.3956696166
H	-3.0658566723	0.729723747	-0.9675337718
C	2.5683495307	0.9049647589	0.4512707824
H	1.0592008462	2.390543055	0.7391413243
C	1.8815182359	-1.1432189045	-0.6263265385
C	-0.4776850658	-1.4353969159	-1.3179183556
H	-2.5418199463	-1.5275330076	-1.8360868499
C	2.8647221049	-0.3811064723	-0.0463674815
H	3.3497095561	1.4955898004	0.9077837205
H	2.1062599123	-2.1309325032	-1.0057504374

H	-0.247144321	-2.4222250596	-1.6938603028
H	3.871416505	-0.7665432111	0.032059084
C	-1.4138064055	-0.7359037649	2.3559675974
O	-0.940310694	-1.7403095364	2.1435219241

34

1-naphthol-DMF complex edge

C	-3.219229993	-0.1771915796	-1.1151708449
C	-3.9106036341	0.9191157279	-0.7175210914
C	-3.9106102739	0.9191174662	0.7175120894
C	-3.2192410532	-0.1771893147	1.1151710045
O	-2.7915783315	-0.8658927567	2.9501e-6
H	-4.3662864963	1.6434339042	-1.3687422564
H	-4.3662990968	1.6434373635	1.368727236
O	0.0099509427	-1.4161911079	8.0006e-6
H	-0.9445640185	-1.2558528512	9.78e-6
C	0.6769597938	-0.2299726715	5.5695e-6
C	2.0981906228	-0.3054850959	-1.629e-7
C	0.0415666584	0.992240675	9.593e-6
C	2.7784782213	-1.5452871389	-4.4448e-6
C	2.8478293832	0.9073221326	-1.4563e-6
C	0.7982113325	2.182258656	8.0949e-6
H	-1.0378866226	1.0464979915	1.44007e-5
C	4.1512999259	-1.5858665461	-9.893e-6
H	2.1986285783	-2.4553915962	-3.5562e-6
C	4.2611899976	0.8252409838	-6.8104e-6
C	2.1686977513	2.1501551825	2.8056e-6

H	0.2775284426	3.1296229963	1.15756e-5
C	4.8993028497	-0.3900512357	-1.10212e-5
H	4.663144374	-2.5377486739	-1.32957e-5
H	4.8328943425	1.7437483044	-7.643e-6
H	2.7445085315	3.0651657891	1.9127e-6
H	5.9793707997	-0.4335477586	-1.52702e-5
C	-2.835324538	-0.7444161183	-2.4285923403
H	-3.2320237499	-1.7516057328	-2.5561069756
H	-1.7511399962	-0.7955940574	-2.5345829579
H	-3.2275504125	-0.1160172284	-3.223902433
C	-2.8353479352	-0.7444103993	2.4285975103
H	-3.2320485888	-1.7515995384	2.5561108269
H	-3.2275813842	-0.1160094826	3.2239022503
H	-1.7511644234	-0.795588289	2.5345988533

21

1-naphthol-N2 complex face

O	-1.3081096143	2.3753655107	0.1874458585
H	-2.2418468421	2.5831681	0.0966721372
C	-1.0792523256	1.1277573228	-0.3165050034
C	0.2563923907	0.6499093417	-0.2352796772
C	-2.0709889316	0.3553238771	-0.8727877008
C	1.292510522	1.4177130923	0.344451854
C	0.5424644887	-0.6508246388	-0.7433369855
C	-1.7731983713	-0.9287830103	-1.3724289702
H	-3.0839174848	0.7341616242	-0.9242969066
C	2.5687653729	0.9162482174	0.4189053677

H	1.0619219296	2.3997464771	0.7276831879
C	1.8699851565	-1.132490589	-0.6497305322
C	-0.4974438196	-1.4265303278	-1.3117320165
H	-2.5677151679	-1.5203646684	-1.8043665678
C	2.8599516486	-0.3695363168	-0.0824371367
H	3.3555641949	1.50800524	0.864492915
H	2.0907093156	-2.1198024073	-1.0325365568
H	-0.270433748	-2.4126165457	-1.6917455033
H	3.8680152663	-0.7538949637	-0.0174129579
N	-1.3196518097	-0.8817165446	2.3676560563
N	-0.8669421709	-1.8497807909	2.1103751383

28

1-naphthol-furan complex edge

C	-3.6607138522	-0.4756304599	-1.0995390787
C	-4.4124126884	0.5843399564	-0.7164108882
C	-4.4124262417	0.5843126029	0.7164381061
C	-3.6607267788	-0.475666775	1.0995399584
O	-3.1909922187	-1.1418345914	-7.8608e-6
H	-4.9077753233	1.2801211271	-1.3687685154
H	-4.907801027	1.2800689194	1.3688128769
H	-3.3701768019	-0.8795260668	2.0505963273
H	-3.3701520422	-0.8794576591	-2.0506054634
O	-0.2962975576	-1.5322412158	5.3293e-6
H	-1.2543094064	-1.4102457739	2.4678e-6
C	0.3219713742	-0.3195187512	6.53e-8
C	1.7447360014	-0.3362599818	1.0891e-6

C	-0.3636179198	0.8744987911	-6.5568e-6
C	2.4763828786	-1.5464578374	8.1006e-6
C	2.4426879156	0.9070453677	-5.3453e-6
C	0.3414878608	2.0954893718	-1.2786e-5
H	-1.4447213033	0.8799886169	-7.157e-6
C	3.8496329646	-1.5292536206	8.8209e-6
H	1.9354673895	-2.4802142588	1.29801e-5
C	3.8582608153	0.8843604045	-4.6432e-6
C	1.7121762249	2.1203587235	-1.23242e-5
H	-0.2179874844	3.0204504178	-1.81575e-5
C	4.5467605356	-0.303062387	2.3278e-6
H	4.4009994441	-2.4587858578	1.44064e-5
H	4.3909096756	1.8260415873	-9.669e-6
H	2.249262292	3.0586000277	-1.73141e-5
H	5.6276962734	-0.3012086779	2.9037e-6

28

1-naphthol-furan complex OH-pi

C	3.7446812865	0.633707037	0.9939593026
C	3.6306114816	-0.6949656754	1.24533813
C	3.7268557158	-1.350765018	-0.0258021206
C	3.8908673296	-0.3656379241	-0.9485329346
O	3.9058483234	0.8522606458	-0.3420160898
H	3.4903781855	-1.1529868197	2.2073192426
H	3.6918569194	-2.4075279058	-0.2215385068
H	4.0110276224	-0.3625366988	-2.0153261944
H	3.7269037494	1.5095232984	1.6140351236

O	0.4758744306	-1.3095536898	0.0398436388
H	1.4140800423	-1.108913464	-0.0669770179
C	-0.2534478548	-0.172059611	-0.1272198833
C	-1.6610209513	-0.292063138	0.0376981266
C	0.3154636292	1.0411071515	-0.4396039887
C	-2.2735269823	-1.5255744824	0.3586371605
C	-2.4677752608	0.8719924537	-0.1275195077
C	-0.4957877548	2.1831202827	-0.5994052749
H	1.3866360606	1.1179127309	-0.5652938177
C	-3.6360030938	-1.6067837083	0.5106541424
H	-1.6510499623	-2.3985759224	0.4810304171
C	-3.8684755769	0.7486723611	0.0373209982
C	-1.8563970236	2.108699871	-0.448115528
H	-0.0278177506	3.1258876723	-0.8457054466
C	-4.4405301253	-0.4596016235	0.3485096437
H	-4.0962917654	-2.5533960771	0.7557587854
H	-4.4838030209	1.6297242173	-0.0873896476
H	-2.4760473724	2.985888579	-0.5717501048
H	-5.5119012811	-0.5350145422	0.4704253519

References

1 Goerigk, L.; Grimme, S. Efficient and Accurate Double-Hybrid-Meta-GGA Density Functionals—Evaluation with the Extended GMTKN30 Database for General Main Group Thermochemistry, Kinetics, and Noncovalent Interactions. *J. Chem. Theory Comput.* **2010**, *7*, 291–309.

2 Grimme, S.; Antony, J.; Ehrlich, S.; Krieg, H. A consistent and accurate ab initio

parametrization of density functional dispersion correction (DFT-D) for the 94 elements H-Pu. *J. Chem. Phys.* **2010**, *132*, 154104.

3 Grimme, S.; Ehrlich, S.; Goerigk, L. Effect of the damping function in dispersion corrected density functional theory. *J. Comput. Chem.* **2011**, *32*, 1456–1465.

4 Weigend, F.; Ahlrichs, R. Balanced basis sets of split valence, triple zeta valence and quadruple zeta valence quality for H to Rn: Design and assessment of accuracy. *Phys. Chem. Chem. Phys.* **2005**, *7*, 3297–3305.

5 Schwilk, M.; Ma, Q.; Köppl, C.; Werner, H.-J. Scalable Electron Correlation Methods. 3. Efficient and Accurate Parallel Local Coupled Cluster with Pair Natural Orbitals (PNO-LCCSD). *J. Chem. Theory Comput.* **2017**, *13*, 3650–3675.

6 Ma, Q.; Schwilk, M.; Köppl, C.; Werner, H.-J. Scalable Electron Correlation Methods. 4. Parallel Explicitly Correlated Local Coupled Cluster with Pair Natural Orbitals (PNO-LCCSD-F12). *J. Chem. Theory Comput.* **2017**, *13*, 4871–4896.

7 Ma, Q.; Werner, H.-J. Scalable Electron Correlation Methods. 5. Parallel Perturbative Triples Correction for Explicitly Correlated Local Coupled Cluster with Pair Natural Orbitals. *J. Chem. Theory Comput.* **2017**, *14*, 198–215.

8 Ma, Q.; Werner, H.-J. Explicitly correlated local coupled-cluster methods using pair natural orbitals. *WIREs Comput. Mol. Sci.* **2018**, *8*, e1371.

9 Ma, Q.; Werner, H.-J. Accurate Intermolecular Interaction Energies Using Explicitly Correlated Local Coupled Cluster Methods [PNO-LCCSD(T)-F12]. *J. Chem. Theory Comput.* **2019**, *15*, 1044–1052.

10 Peterson, K. A.; Adler, T. B.; Werner, H.-J. Systematically convergent basis sets for explicitly correlated wavefunctions: The atoms H, He, B–Ne, and Al–Ar. *J. Chem. Phys.* **2008**, *128*, 084102.

11 Werner, H.-J.; Knowles, P. J.; Knizia, G.; Manby, F. R.; Schütz, M. Molpro: a general-purpose quantum chemistry program package. *Wiley Interdiscip. Rev. Comput. Mol. Sci.* **2012**, *2*, 242–253.

12 Werner, H.-J.; Knowles, P. J.; Manby, F. R.; Black, J. A.; Doll, K.; Heßelmann, A.; Kats, D.; Köhn, A.; Korona, T.; Kreplin, D. A.; Ma, Q.; Miller, T. F.; Mitrushchenkov, A.; Peterson, K. A.; Polyak, I.; Rauhut, G.; Sibaev, M. The Molpro quantum chemistry package. *J. Chem. Phys.* **2020**, *152*, 144107.

13 Werner, H.-J.; Knowles, P. J.; Celani, P.; Györffy, W.; Hesselmann, A.; Kats, D.; Knizia, G.; Köhn, A.; Korona, T.; Kreplin, D.; Lindh, R.; Ma, Q.; Manby, F. R.; Mitrushenkov, A.; Rauhut, G.; Schütz, M.; Shamasundar, K. R.; Adler, T. B.; Amos, R. D.; Bennie, S. J.; Bernhardsson, A.; Berning, A.; Black, J. A.; Bygrave, P. J.; Cimiraglia, R.; Cooper, D. L.; Coughtrie, D.; Deegan, M. J. O.; Dobbyn, A. J.; Doll, K.; Dornbach, M.; Eckert, F.; Erfort, S.; Goll, E.; Hampel, C.; Hetzer, G.; Hill, J. G.; Hodges, M.; Hrenar, T.; Jansen, G.; Köppl, C.; Kollmar, C.; Lee, S. J. R.; Liu, Y.; Lloyd, A. W.; Mata, R. A.; May, A. J.; Mussard, B.; McNicholas, S. J.; Meyer, W.; Miller III, T. F.; Mura, M. E.; Nicklass, A.; O'Neill, D. P.; Palmieri, P.; Peng, D.; Peterson, K. A.; Pflüger, K.; Pitzer, R.; Polyak, I.; Reiher, M.; Richardson, J. O.; Robinson, J. B.; Schröder, B.; Schwilk, M.; Shiozaki, T.; Sibaev, M.; Stoll, H.; Stone, A. J.; Tarroni, R.; Thorsteinsson, T.; Toulouse, J.; Wang, M.; Welborn, M.; Ziegler, B. MOLPRO, version 2022.2, a package of ab initio programs. see https://www.molpro.net.

14 Hill, J. G.; Peterson, K. A.; Knizia, G.; Werner, H.-J. Extrapolating MP2 and CCSD explicitly correlated correlation energies to the complete basis set limit with first and second row correlation consistent basis sets. *J. Chem. Phys.* **2009**, *131*, 194105.

15 Wuttke, A.; Mata, R. A. Visualizing dispersion interactions through the use of local orbital spaces. *J. Comput. Chem.* **2016**, *38*, 15–23.

16 Werner, H.-J.; Knizia, G.; Krause, C.; Schwilk, M.; Dornbach, M. Scalable Electron Correlation Methods I.: PNO-LMP2 with Linear Scaling in the Molecular Size and Near-Inverse-Linear Scaling in the Number of Processors. *J. Chem. Theory and Comput.* **2015**, *11*, 484–507.
